# Candle Soot-Based Electrosprayed Superhydrophobic Coatings for Self-Cleaning, Anti-Corrosion and Oil/Water Separation

**DOI:** 10.3390/ma15155300

**Published:** 2022-08-01

**Authors:** Yuting Zhang, Tingping Lei, Shuangmin Li, Xiaomei Cai, Zhiyuan Hu, Weibin Wu, Tianliang Lin

**Affiliations:** 1 College of Mechanical Engineering and Automation, Huaqiao University, Xiamen 361021, China; ytzhang@stu.hqu.edu.cn (Y.Z.); 1711113012@stu.hqu.edu.cn (S.L.); hzy1999@mail.ustc.edu.cn (Z.H.); ltl@hqu.edu.cn (T.L.); 2 Fujian Provincial Key Laboratory of Special Energy Manufacturing, Huaqiao University, Xiamen 361021, China; 3 School of Science, Jimei University, Xiamen 361021, China; 201921145023@jmu.edu.cn

**Keywords:** superhydrophobic coatings, electrospraying, candle soot, self-cleaning, anti-corrosion, oil/water separation

## Abstract

The interest in candle soot (CS)-based superhydrophobic coatings has grown rapidly in recent years. Here, a simple and low-cost process has been developed for the fabrication of CS-based superhydrophobic coatings through electrospraying of the composite cocktail solution of CS and polyvinylidene fluoride (PVDF). Results show that the superhydrophobicity of the coating closely relates to the loading amount of CS which results in coatings with different roughnesses. Specifically, increasing the CS amount (not more than 0.4 g) normally enhances the superhydrophobicity of the coating due to higher roughness being presented in the produced microspheres. Further experiments demonstrate that the superhydrophobicity induced in the electrosprayed coating results from the synergistic effect of the cocktail solution and electrospray process, indicating the importance of the coating technique and the solution used. Versatile applications of CS-based superhydrophobic coatings including self-cleaning, anti-corrosion and oil/water separation are demonstrated. The present work provides a convenient method for the fabrication of CS-based superhydrophobic coatings, which is believed to gain great interest in the future.

## 1. Introduction

Candle soot (CS), traditionally deemed as a source of unwanted air pollution, has received increasing attention in recent years [1,2,3]. Fresh CS collected from the inner flame is superhydrophobic (water contact angle, WCA ≥ 150°) [4], but pristine CS is fragile and oxidation during aging causes the soot to become hydrophilic [5]. To obviate these problems, researchers have proposed numerous approaches that can be classified into the following three major categories: (1) substrate pretreatment before CS deposition, either through coating polydimethylsiloxane (PDMS) mixtures [6,7,8] or paraffin wax [9] on the raw substrate, or making the substrate much rougher (e.g., via electrodeposition [10] or other methods [11,12]); (2) reinforcement after CS deposition, mainly via covering the CS layer with PDMS mixtures [13,14] or some specific polymer solutions [15]; and (3) mixing CS with polymer for solution deposition of superhydrophobic coatings [16,17,18].

Among them, the method of mixing CS with polymer is much simpler and more flexible in the selection of both polymer materials and deposition methods. The materials, such as PDMS, polyurethane (PU), and polyvinylidene fluoride (PVDF), and the deposition methods, such as spray coating, spin coating, and gelation technique, can be used for making superhydrophobic polymer/CS composite coatings. Literature suggests that spray coating has been widely adopted by many researchers. Sutar et al. reported the use of the spray technique to deposit PDMS/CS composite for self-cleaning superhydrophobic coating [19]. Li et al. employed the spray technique to fabricate superhydrophobic PU/CS coatings for oil–water separation [16]. The spray coating was also used for the scalable fabrication of superhydrophobic PVDF/SiO_2_ membranes for gravitational water-in-oil emulsion separation [20]. Compared with the conventional spray coating, electrospraying (electrohydrodynamic spraying, a process utilizing the electric field alone rather than additional mechanical energy to generate fine droplets with charge) allows for better control and higher deposition efficiency of the atomized charged droplets to self-disperse in smaller sizes [21]. The technique of electrospraying has been intensively studied in the synthesis of micro/nano materials [22,23] and mass spectrometry [24,25]. To the best of our knowledge, electrospraying of CS-based superhydrophobic coatings are seldom reported, although recent works utilized this technique to deposit functionalized CS particles and carbonaceous nanoparticle layers [26,27].

In this work, CS-based superhydrophobic coatings were demonstrated through electrospraying of the composite cocktail solution of CS mixed with PVDF (a commercially available fluoropolymer with low surface energy, 25 dynes/cm). The synergetic effect and the main factors influencing the hydrophobicity of the coatings were investigated, and the typical applications in self-cleaning, anti-corrosion, and oil/water separation were presented.

## 2. Experimental

Polyvinylidene fluoride (*M*_W_ ~ 625,000) in powder form was purchased from Shanghai Sensure Chemical Co., Ltd. (Xi’an, China). Analytically pure N, N-dimethylformamide (DMF), and acetone were obtained from Sinopharm Chemical Reagent Co., Ltd. (Shanghai, China) and used as received. Candles and substrates including glass slides, stainless steel and copper meshes, cotton fabrics, printing paper, iron sheets, wood panels, and cobblestones were purchased from local supermarkets. The superhydrophobic CS was collected from the middle zone of candle flame as described previously [18,28]. Specifically, by placing a stainless steel plate above the outer flame of the burning candle for 1 min, a thick layer of CS particles was obtained on the plate, which could be further scraped and transferred for use.

A standard electrospray apparatus was utilized to deposit PVDF-CS composite coatings, where the solution flow rate was controlled by a precision syringe pump (NE-300, New Era Pump Systems Inc., Farmingdale, NY, USA). The cocktail solution was prepared by continuously stirring the mixture of 0.3 g CS (if not stated otherwise) with a PVDF solution that was formed by dissolving 0.2 g PVDF powders in 6 mL DMF and 4 mL acetone. Before the electrospraying process, the cocktail solution was degassed to remove air bubbles. During the electrospraying experiments, the applied voltage was set at 7.0 kV, the solution feed rate was 500 μL/h, and the needle tip-to-collector distance was 10 cm. All experiments were performed under an air atmosphere with a relative humidity between 55 and 60%.

The surface morphology of the as-prepared samples was observed by a scanning electron microscope (SEM, SU70 and SU5000, Hitachi, Tokyo, Japan). The hydrophobicity characterization was conducted on a contact angle analyzer (JC2000D3, Shanghai Zhongchen Digital Technology Equipment Co., Ltd., Shanghai, China) by placing water droplets of 9 μL on the coatings. The contact angle data were figured out based on the ellipse fitting method, and the final result was averaged from five measurements per specimen.

The self-cleaning property was evaluated by sprinkling the chalk powder on a tilted coated substrate and slowly dropping water droplets on it. The oil/water separation was performed by pouring oil/water mixtures into the coated copper mesh that was tailed into a “Taylor cone” container, where hexadecane and chloroform were used as light and heavy oils, respectively. The anti-corrosion was tested by soaking stainless steel mesh (both uncoated and coated) in concentrated HCl solution.

## 3. Results and Discussion

**Figure 1:**
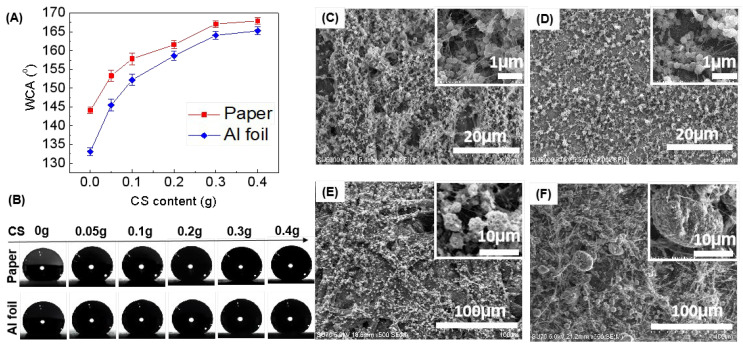
Effect of CS content on the hydrophobicity and surface morphology of electrosprayed paper and Al foil substrates: (**A**) water contact angle (WCA) varied with CS content; (**B**) typical WCA photos of the curves from (**A**) (the arrow indicates the increasing direction of CS content); (**C**–**F**) SEM images of electrosprayed samples with CS contents of 0 g and 0.3 g on paper (**C**,**E**) and Al foil (**D**,**F**), respectively (insets are the enlarged images of each samples).

Figure 1 illustrates the hydrophobicity and morphology of the electrosprayed coatings on paper and Al foil substrates with different loading amounts of superhydrophobic CS. As shown in Figure 1A, with the loading of CS into PVDF solution, all PVDF/CS composite coatings either on paper or on the Al foil substrate show visible improvement in hydrophobicity (as compared with the pure PVDF coatings), although there are some differences. When 0.05 g CS is loaded, the coated paper already becomes superhydrophobic (average WCA ~ 153°) and the coated Al foil sample is also close to superhydrophobic (average WCA ~ 145°). Further increasing the CS loading allows the composite coatings to further improve in superhydrophobicity. The highest WCA obtained in our repeated experiments was about 168° for the coated paper when a higher amount of CS (e.g., 0.4 g) was loaded. The representative WCA photos of the corresponding curves in Figure 1A are demonstrated in Figure 1B, where the arrow indicates the increasing direction of CS content.

Surface morphologies of the pure PVDF coatings and the typical PVDF/CS coatings on both substrates are shown in Figure 1C–F. It is observed that electrosprayed coatings from pure PVDF solution are chiefly composed of smooth nanospheres (200~300 nm), although with some ultrafine fibers (Figure 1C,D). In contrast, the coatings electrosprayed with the PVDF/CS cocktail solution present rough microspheres in large numbers (Figure 1E,F). That is why the electrosprayed coatings using the low surface energy materials of PVDF and CS show better hydrophobicity than the coatings using the pure PVDF. On the other hand, from the results shown in Figure 1A, it is reasonable to infer that increasing the CS loading enhances the roughness of the coatings, and therefore a better hydrophobicity is normally obtained. However, it should be noted that when the loading amount of CS reaches 0.4 g, continuous loading has little contribution to the enhancement of hydrophobicity.

**Figure 2:**
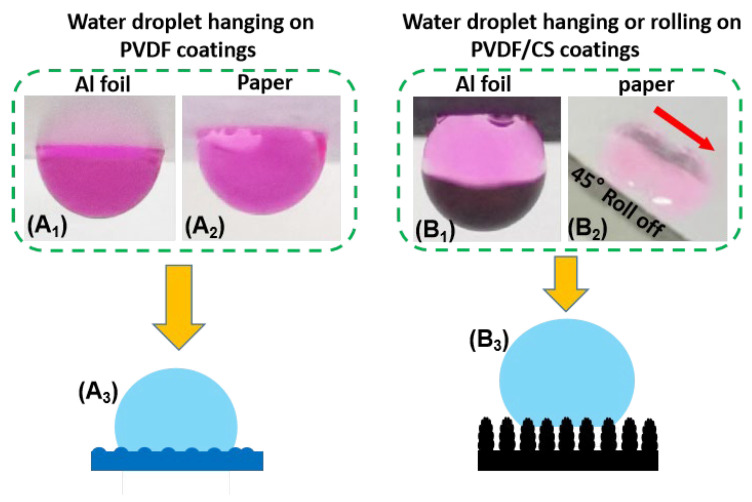
Wetting behavior and regime of the electrosprayed coatings: (**A_1_**,**A_2_**) and (**B_1_**) water droplets hanging on the coatings at a tilt of 180°; (**B_2_**) water droplet rolling off the coatings at a tilt of 45°; (**A_3_**,**B_3_**) schematics of the Wenzel model and the combined Cassie–Baxter/Wenzel model for the corresponding coatings, respectively.

Besides the hydrophobicity, the “stickiness” of the coatings is also significant from the application point of view. Figure 2A_1_,A_2_ shows that pure PVDF coatings (either on the Al foil or paper substrate) are very sticky, as evidenced by the observation of semi-spherical water droplets on the coatings at a tilt of 180°. This highly sticky hydrophobic coating was reported to be the result of the pseudo-hydrogen bonding effect of the polarized C-H bonds in each repeating unit of PVDF polymer chains [29]. Unlike pure PVDF coatings, PVDF/CS composite coatings show different stickiness behaviors for the substrate. The composite coatings on paper allow the water droplet to roll off at a tilt of 45° (Figure 2B_2_), whereas the coatings on the Al foil still show water-stickiness (Figure 2B_1_) although not so strong as the pure PVDF coatings in Figure 2A_1_,A_2_. Thus, the loading of CS into PVDF solution during the electrospraying process not only enhances the coating hydrophobicity, but also reduces the “stickiness” of the coatings, which is useful for potential applications in the field of self-cleaning and anti-corrosion.

According to the surface morphology of the coatings (Figure 1C–F) and their wetting behavior (hydrophobicity (Figure 1A) and “stickiness” (Figure 2A_1_,A_2_,B_1_,B_2_), it is reasonable to state that hydrophobic electrosprayed PVDF coatings follow the total wetting Wenzel state (Figure 2A_3_), and superhydrophobic electrosprayed PVDF/CS coatings follow the combined Cassie–Baxter/Wenzel state (Figure 2B_3_). It should be emphasized that the superhydrophobicity presented in the electrosprayed coatings is attributed to the synergistic effect of using the PVDF/CS cocktail solution and the process employed. As shown in Figure 1, the electrosprayed coatings from pure PVDF solution (without CS loading) are hydrophobic rather than superhydrophobic, since the assembly of smooth nanospheres can not make the coating rough enough to achieve superhydrophobicity [30]. In contrast, the electrosprayed coatings with the PVDF/CS cocktail solution easily become superhydrophobic due to the formation of numerous rough microspheres that probably result from incompatible CS nanoparticles and PVDF macromolecules bonded together at their contact points or interfaces (since CS and PVDF are of completely different solubilities in DMF). However, it is noted that the PVDF/CS coatings fabricated by spin coating or solution casting show poorer hydrophobicity than the electrosprayed PVDF coatings. In this regard, electrospraying is more powerful in the fabrication of (super)hydrophobic coatings.

**Figure 3:**
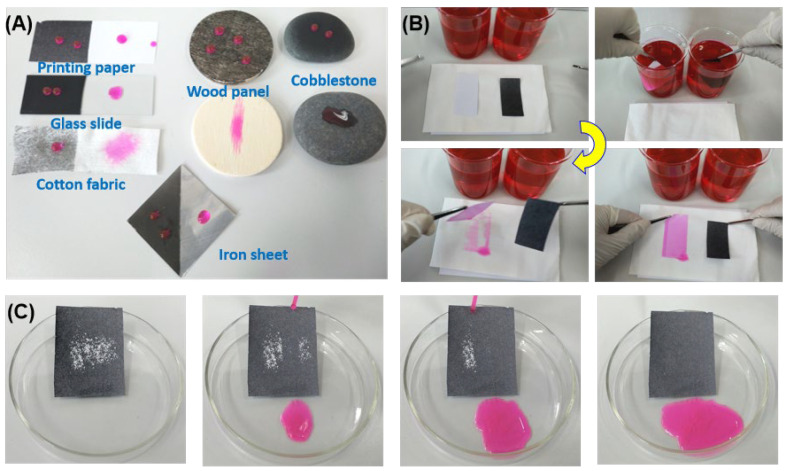
(**A**) Wetting behaviors of various substrates with/without electrosprayed PVDF/CS coatings by placing some 12 μL water droplets (dyed red) on them; (**B**) wetting behaviors of double-faced superhydrophobic printing paper (coated with the same coatings as (**A**)) and the uncoated paper, when they are first immersed into the red water and then placed on the cotton; (**C**) snapshots showing the self-cleaning property of the coated printing paper by slowly dropping water droplets on the surface which has been sprinkled with the chalk powder.

In the electrospraying process, the solution is highly charged and undergoes Coulomb fission and breaks into tiny self-dispersing droplets [21]. As the solvent evaporates, deposits (coatings) of different morphologies and roughnesses are produced on the target substrate depending on the solution parameters (solution composition, solvent evaporation rate, etc.) and the processing parameters (applied voltage, solution feed rate, and the needle tip-to-collector distance, etc.). Thus, the coatings electrosprayed using (cocktail) solution with low surface energy are hydrophobic or superhydrophobic, as shown in Figure 1. This simple process also allows for superhydrophobic deposits on any solid substrates of various geometrical configurations [31,32]. The representative substrates (including glass slide, cotton fabric, printing paper, iron sheet, wood panel, and cobblestone) with PVDF/CS coatings show excellent water repellency as compared with the untreated substrates (Figure 3A). Moreover, a double-faced superhydrophobic printing paper was fabricated via electrospraying with PVDF/CS composites on both sides of the paper. As shown in Figure 3B, the coated paper is perfectly clean as it is taken out from the red water, whereas the uncoated paper is completely wetted by the water and leaves the stains on the cotton.

Nowadays, self-cleaning plays a significant role in various applications (such as buildings, solar panels and wind shields), in that keeping the surface self-clean means that no additional cost is required for the maintenance, and the service life is also increased. Figure 3C demonstrates the self-cleaning performance of electrosprayed PVDF/CS-coated printing paper by randomly sprinkling chalk powder (used as the contaminant) on the coated substrate that was tilted 15°. A red water droplet (12 μL) was slowly dropped on the substrate from a height of 1.5 cm above the surface. The powder particles are taken away with water droplets as they slide down the surface (Figure 3C), suggesting the self-cleaning nature.

**Figure 4:**
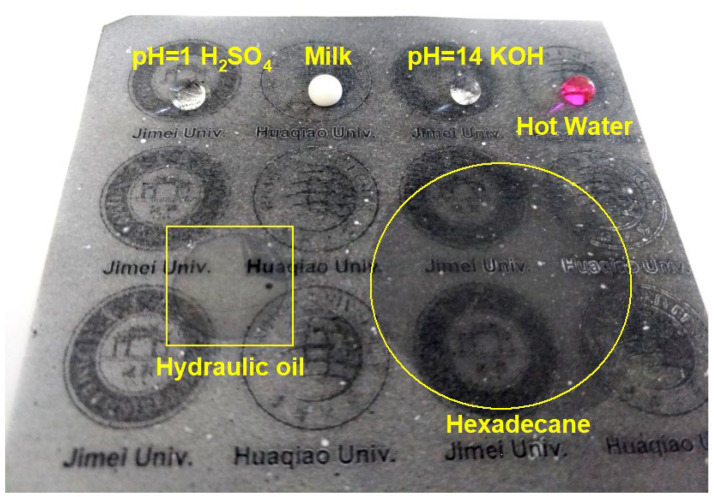
Super big droplets (c.a. 0.15 mL) of various liquids on the printing paper that is coated with electrosprayed PVDF/CS composites. Details are indicated in the figure, where the temperature for the hot water is about 85 °C.

The wetting property of the electrosprayed PVDF/CS coating was further investigated by subjecting it to droplets of various liquids. As shown in Figure 4, all aqueous droplets (H_2_SO_4_, KOH, and hot water) exhibit perfect spherical shapes, except for the milk droplet which slightly lost its sphericity (possibly due to the different composition in milk that contains protein, fat, lactose, and minerals besides water [33]), whereas the oil droplets spread over the coated paper. In fact, n-hexadecane (3.36 cP) spreads immediately as it touches the surface, indicating that the coating is also superoleophilic. On the other hand, the long-term superhydrophobicity to concentrated H_2_SO_4_ (pH = 1) and KOH (pH = 14) solutions suggests the feasibility of using the coating under harsh conditions. The above results show the potential for practical use of the coating in the separation of low viscosity oil/water mixtures.

**Figure 5:**
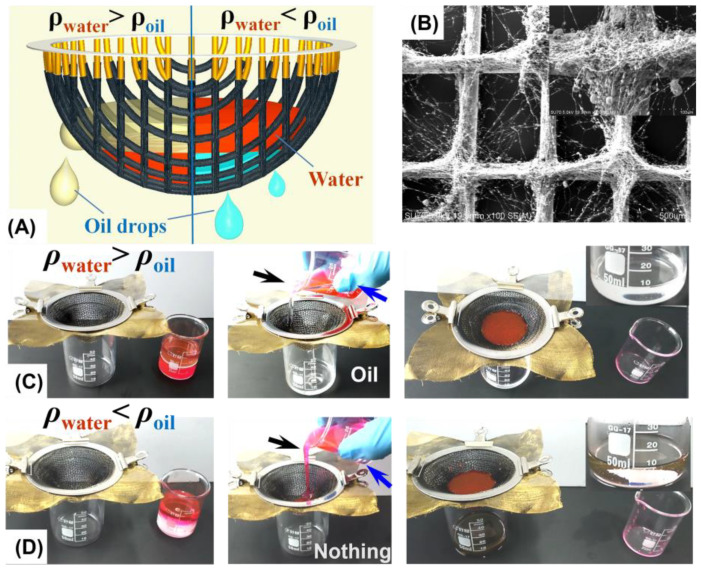
(**A**) Schematics of a superhydrophobic and superoleophilic “Taylor cone”-like 3D mesh container for both light and heavy oil/water separation; (**B**) SEM image of the coated copper mesh with electrosprayed PVDF/CS composites (enlarged in inset); snapshots of the use of the mesh container to separate light oil/water mixtures (**C**) and heavy oil/water mixtures (**D**), where hexadecane and chloroform are used as light and heavy oils, respectively. The water was dyed red.

As was previously pointed out by most publications [28,34,35], the use of superhydrophobic and superoleophilic (or superhydrophilic and underwater superoleophobic) mesh in oil/water separation normally encountered the problem of a water (or oil) barrier that blocks oil (or water) from passing through the membrane. To avoid this problem, we propose a “Taylor cone”-like 3D mesh container that allows both oil and water to touch the container wall, such that the separation of both light (*ρ*_oil_ < *ρ*_water_) and heavy (*ρ*_oil_ > *ρ*_water_) oil/water mixtures can be achieved. As illustrated in Figure 5A, when light oil/water mixtures are loaded into the superhydrophobic and superoleophilic mesh container, the oil (green) can pass the mesh even though water (red) settles down to form the barrier. For the separation of heavy oil/water mixtures, the oil (light blue) can directly pass through the bottom, whereas water (red) is blocked in the container. To validate the proposed model, a superhydrophobic and superoleophilic copper mesh (120) was electrosprayed with PVDF/CS composites, the SEM image of which is shown in Figure 5B. The as-prepared mesh was then sandwiched between two identical structural stainless steel meshes (200) to form the “Taylor cone” container. Light oil/water separation was performed by pouring a mixture of 10 mL hexadecane and 10 mL water into the container, and the separation was successfully finished after a few seconds (Figure 5C). A similar process was successfully conducted for heavy oil/water separation, where chloroform was used as the heavy oil (Figure 5D). By properly pumping out the water accumulated in the container, continuous oil/water separation can be realized for oils of any density [28].

**Figure 6:**
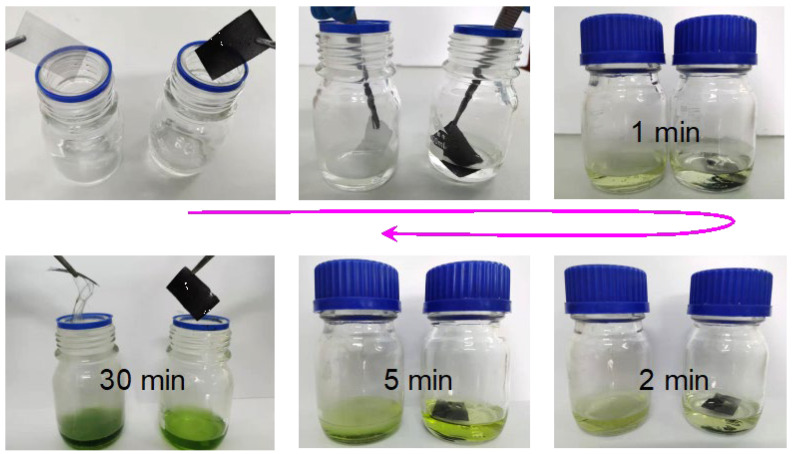
Comparison of anti-corrosive behavior for the uncoated stainless steel mesh and the double-faced superhydrophobic mesh coated with PVDF/CS in concentrated HCl solution.

The anti-corrosion experiment was conducted by immersing the uncoated stainless steel mesh and the double-faced superhydrophobic mesh (coated with PVDF/CS coatings) into the concentrated HCl solution. As shown in Figure 6, the uncoated mesh reacts with the solution more rapidly than the coated mesh, as revealed by darkening of the solution color. After 30 min, both of the meshes were carefully picked up for comparison. The double-faced mesh was nearly intact, whereas the uncoated mesh was badly destroyed, indicating the potential of using the coatings to protect the metal from corrosion. The agglomeration and chemical inertness of CS particles provide PVDF/CS coatings with a rough surface topography and chemical inhomogeneity, allowing the coatings to be in the Cassie–Baxter state to slow down solution erosion [11,36].

## 4. Conclusions

In summary, a simple and low-cost process for the fabrication of CS-based superhydrophobic coating was developed. Electrospraying with CS and PVDF composite cocktail solution can induce numerous rough microspheres in the coating and therefore realize superhydrophobicity. The comparison of the solution and coating methods reveals that the formation of superhydrophobicity is attributed to the synergistic effect of the cocktail solution and electrospraying. Both the CS loading and the substrate influence the superhydrophobicity of the CS-based electrosprayed coatings. The as-prepared CS-based coatings show potential applications in the fields of self-cleaning, oil/water separation, and anti-corrosion.

## Data Availability

The data presented in this study are available in the manuscript.
